# Impact of complete surgical resection on outcome in aggressive non‐Hodgkin lymphoma treated with immunochemotherapy

**DOI:** 10.1002/cam4.3448

**Published:** 2020-09-14

**Authors:** Christine Schmitz, Jan Rekowski, Stefan P. Müller, Navid Farsijani, Bernd Hertenstein, Christiane Franzius, Ulla von Verschuer, Paul La Rosée, Martin Freesmeyer, Stefan Wilop, Thomas Krohn, Aruna Raghavachar, Arnold Ganser, Frank M. Bengel, Gabriele Prange‐Krex, Frank Kroschinsky, Jörg Kotzerke, Aristoteles Giagounidis, Ulrich Dührsen, Andreas Hüttmann

**Affiliations:** ^1^ Klinik für Hämatologie Universitätsklinikum Essen Universität Duisburg‐Essen Essen Germany; ^2^ Institut für Medizinische Informatik, Biometrie und Epidemiologie Universitätsklinikum Essen Universität Duisburg‐Essen Essen Germany; ^3^ Klinik für Nuklearmedizin Universitätsklinikum Essen Universität Duisburg‐Essen Essen Germany; ^4^ Medizinische Klinik I Klinikum Bremen‐Mitte Bremen Germany; ^5^ Zentrum für moderne Diagnostik (Zemodi) Zentrum für Nuklearmedizin und PET/CT Bremen Germany; ^6^ MVZ Hämatologie und Onkologie Essen Germany; ^7^ Klinik für Innere Medizin II Universitätsklinikum Jena Jena Germany; ^8^ Klinik für Nuklearmedizin Universitätsklinikum Jena Jena Germany; ^9^ Klinik für Hämatologie, Onkologie, Hämostaseologie und Stammzelltransplantation Universitätsklinikum Aachen Aachen Germany; ^10^ Klinik für Nuklearmedizin Universitätsklinikum Aachen Aachen Germany; ^11^ Medizinische Klinik 1 Helios Universitätsklinikum Wuppertal Wuppertal Germany; ^12^ Klinik für Hämatologie, Hämostaseologie, Onkologie und Stammzelltransplantation Medizinische Hochschule Hannover Hannover Germany; ^13^ Klinik für Nuklearmedizin Medizinische Hochschule Hannover Hannover Germany; ^14^ Onkologische Gemeinschaftspraxis Dresden Germany; ^15^ Medizinische Klinik I Universitätsklinikum Carl Gustav Carus Dresden Germany; ^16^ Klinik für Nuklearmedizin Universitätsklinikum Carl Gustav Carus Dresden Germany; ^17^ Klinik für Onkologie und Hämatologie Helios St. Johannes Klinik Duisburg Germany; ^18^ Present address: Klinik für Innere Medizin II Villingen‐Schwenningen Germany; ^19^ Present address: Radiologie Aachen Land Würselen Germany; ^20^ Present address: Klinik für Onkologie Marien Hospital Düsseldorf Germany

**Keywords:** Lymphoma, , B‐cell, lymphoma, , T‐cell, positron emission tomography, prognosis, surgical resection

## Abstract

**Background:**

Surgical resection is considered to be of purely diagnostic value in aggressive lymphoma. Evidence for an impact on outcome is scant and restricted to retrospective observations.

**Methods:**

In the “Positron Emission Tomography‐guided Therapy of Aggressive non‐Hodgkin Lymphomas” (PETAL) trial, patients with a negative baseline positron emission tomography (PET) scan were documented in a prospective observational substudy. Baseline PET‐negative patients with the absence of lymph node enlargement on computed tomography and a negative bone marrow biopsy were considered to have undergone complete lymphoma resection.

**Results:**

Eighty‐two of 1,041 patients (7.9%) had a negative baseline PET scan, and 67 were included in this analysis. All were treated with cyclophosphamide, doxorubicin, vincristine, and prednisone (CHOP), plus rituximab for CD20‐positive lymphomas. Among 52 patients with diffuse large B‐cell lymphoma (DLBCL), 48 had completely resected disease. Their outcome tended to be better than that of 115 baseline PET‐positive stage I DLBCL patients treated in the main part of the PETAL trial (2‐year progression‐free survival 92.7% [95% confidence interval 84.7‐100] versus 88.4% [82.5‐94.3], *P* = .056; 2‐year overall survival 92.7% [84.7‐100] versus 93.7% [89.2‐98.2], *P* = .176), but this was restricted to patients below the age of 60 years (2‐year progression‐free survival 100% versus 92.2% [84.8‐99.6], *P* = .031; 2‐year overall survival 100% versus 95.9% [90.2‐100], *P* = .075). In peripheral T‐cell lymphoma, eight of 11 patients had completely resected disease. In contrast to DLBCL, complete resection was not associated with improved outcome compared to the control.

**Conclusion:**

Young patients with early stage DLBCL may benefit from complete lymphoma resection prior to immunochemotherapy.

## Graphical abstract

In stage I diffuse large B‐cell lymphoma outcome was better after complete than incomplete lymphoma resection. In contrast to B‐cell lymphoma, no benefit for complete resection was seen in T‐cell lymphoma.

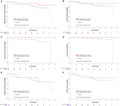

## 1 INTRODUCTION

In aggressive non‐Hodgkin lymphoma, surgical lymph node resection is considered to be of purely diagnostic value. It is increasingly being replaced by core needle biopsy which, while being less invasive, often allows the pathologist to establish a firm diagnosis.^1^ Unlike solid tumors, lymphoma treatment is almost exclusively based on systemic therapy.

Modern imaging techniques, such as [18]fluorodeoxyglucose (FDG) positron emission tomography (PET), have highlighted the overwhelming impact of tumor burden on outcome.^2^ We recently showed that four of the five factors of the International Prognostic Index (Ann Arbor stage, extranodal disease, performance status, lactate dehydrogenase)^3^ were mere surrogates of total metabolic tumor volume as measured by FDG‐PET.^4^ Given the relationship between tumor mass and prognosis it is tempting to speculate that surgical debulking prior to systemic therapy may improve outcome.

The impact of complete surgical resection on outcome was investigated in a prospective substudy of the “Positron Emission Tomography‐guided Therapy of Aggressive non‐Hodgkin Lymphomas” (PETAL) trial. The main part of the trial set out and failed to improve outcome by adjusting treatment‐to‐treatment response as measured by interim PET after two cycles of cyclophosphamide, doxorubicin, vincristine, and prednisone (CHOP; plus rituximab [R] in CD20‐positive lymphomas).^5^ Because interim PET was evaluated by comparing the maximum standardized uptake values at baseline and interim staging, patients with a negative baseline PET scan were excluded from the main part of the trial. Instead, they took part in an observational substudy in which therapy was delivered at the treating physician's discretion.

In the present report, we compare patients with aggressive B‐cell and T‐cell lymphomas and a negative baseline PET scan with baseline PET‐positive patients who were treated in the main part of the PETAL trial. In the majority of patients, a negative baseline PET scan was the result of complete lymphoma resection.

## 2 METHODS

### 2.1 Patients

The PETAL trial (EudraCT 2006‐001641‐33, ClinicalTrials.gov NCT00554164) was a multicenter, prospective, randomized, controlled study in which patients with newly diagnosed aggressive B‐cell or T‐cell non‐Hodgkin lymphoma were eligible to participate.^5^ Patients without FDG‐avid disease at baseline PET scanning were excluded from the main part of the trial. Their disease was managed at the treating physician's discretion, and the course was prospectively documented in an observational sub‐study. The trial was approved by the Federal Institute for Drugs and Medical Devices (Germany) and the ethics committees of all participating sites. All patients gave written informed consent.

### 2.2 Imaging procedures

Baseline PET scanning was uniformly performed before therapy (including glucocorticosteroids).^5^ Patients with a negative scan were reanalyzed by review of the original PET and computed tomography (CT) reports. A negative PET scan was defined by the absence of nonphysiological FDG uptake according to International Harmonization Project criteria.^6^ CT scans were reassessed by applying the Lugano Classification where nodal and extranodal manifestations are defined by maximum diameters exceeding 1.5 cm or 1.0 cm, respectively.^7^ Since surgical accessibility may influence resection, superficial and deep lymphoma locations were analyzed separately. Superficial locations included cervical, periclavicular, axillary and inguinal lymph nodes, skin, parotid gland, tonsils, vagina/uterine cervix, and testicles. All other locations were considered deep.

Absence of detectable lesions on both CT and PET together with a negative bone marrow biopsy was interpreted as completely resected disease. When bone marrow biopsy was not performed, we assumed it to be negative because this is the case in >95% of early stage aggressive lymphomas.^8^ Measurable disease on baseline CT, but not PET, was suggestive of a tumor lacking increased glucose uptake (subsequently referred to as “FDG‐negative lymphoma”). Metabolic tumor volume was determined by using the 41% maximum standardized uptake value thresholding method.^4^

### 2.3 Statistical analysis

Data are described as median and range for continuous variables and absolute and relative frequencies for categorical variables. Fisher's exact test was used to compare frequencies. Complete remission and overall response rates were defined as the end‐of‐treatment staging fraction of patients with complete remission or complete and partial remission combined, respectively. Overall survival defined as time from baseline staging to death from any cause, and progression‐free survival defined as time from baseline staging to progression or death from any cause were estimated by the Kaplan‐Meier estimator^9^ and compared by the log‐rank test. Patients with a negative baseline PET scan were compared with patients with a positive baseline PET scan treated in the main part of the PETAL trial. The Cox proportional hazard model^10^ was used for estimation of the hazard ratio and its confidence interval. A Cox regression model was employed to investigate effects on progression‐free survival and overall survival.

## 3 RESULTS

### 3.1 Patients’ baseline characteristics

Of 1,073 patients registered for the PETAL trial, 1,041 underwent baseline investigation by PET and CT. Reasons for exclusion were histological misdiagnosis in 24 patients and scanner failure or premature treatment initiation in four patients each. Eighty‐two of 1,041 patients had a negative baseline PET scan (7.9%). After exclusion of patients with a change of diagnosis after reference pathological review or insufficient follow‐up, a total of 67 patients remained for the substudy of baseline PET‐negative lymphomas (Table 1).

**Table 1:** Baseline characteristics and treatment details

	B‐cell lymphoma N = 56		T‐cell lymphoma N = 11	
Median age at screening, years (range)	58	(27‐79)	59	(20‐78)
Male sex	39	(69.6%)	9	(81.8%)
B symptoms	2	(3.5%)	5	(45.5%)
International Prognostic Index > 2	0	(0.0%)	1	(9.1%)
Age ≥ 60 years	26	(46.4%)	4	(36.4%)
Ann Arbor stage III or IV	1^a^	(1.8%)	2^b^	(18.2%)
Extranodal sites > 1	0		1^c^	(9.1%)
ECOG performance status ≥ 2	0		0	
Lactate dehydrogenase > ULN	7	(12.5%)	3	(27.3%
FDG‐negative lesions on CT scan	3	(5.4%)	2	(18.2%)
Positive bone marrow biopsy^d^	1	(2.7%)	1	(12.5%)
Histological diagnoses				
Diffuse large B‐cell lymphoma	52	(92.8%)		
Follicular lymphoma grade 3b	2	(3.6%)		
Other large B‐cell lymphomas	2	(3.6%)		
Peripheral T‐cell lymphoma, NOS			2	(18.2%)
Anaplastic large‐cell lymphoma, ALK pos.			2	(18.2%)
Anaplastic large‐cell lymphoma, ALK neg.			1	(9.1%)
Enteropathy‐associated T‐cell lymphoma			3	(27.3%)
Angioimmunoblastic T‐cell lymphoma			2	(18.2%)
Unclassifiable T‐cell lymphoma			1	(9.1%)
Treatment				
2 × R‐CHOP + 4 x R‐bendamustine	1	(1.8%)	0	
3 × R‐CHOP ± radiotherapy	2	(3.6%)	0	
4 × (R‐)CHOP^e^	4	(7.1%)	2	(18.2%)
5 × R‐CHOP	3	(5.4%)	0	
6 × (R‐)CHOP^e^	42	(75%)	8	(72.7%)
Unknown	4	(7.1)	1	(9.1)

Abbreviations: ALK, anaplastic lymphoma kinase; CHOP, cyclophosphamide, doxorubicin, vincristine, and prednisone; CT, computed tomography; ECOG, Eastern Cooperative Oncology Group; FDG, [^18^F]fluorodeoxyglucose; neg., negative; NOS, not otherwise specified; pos., positive; R, rituximab; ULN, upper limit of normal.

Data are given as number (%) unless otherwise noted.

^a^ Stage IV due to bone marrow involvement.

^b^ Stage III or IV due to lymph node enlargement or bone marrow and skin involvement, respectively.

^c^ Bone marrow and skin involvement.

^d^ Bone marrow biopsy was performed in 37 B‐cell and eight T‐cell lymphoma patients.

^e^ Use of rituximab (R) was limited to B‐cell lymphomas.

Fifty‐two of 56 patients with B‐cell lymphoma (93%) and eight of 11 patients with T‐cell lymphoma (73%) had no evidence of lymphoma on CT and a negative bone marrow biopsy, consistent with complete lymphoma resection. The remaining patients had nodal enlargement detected by CT, but not PET or a positive bone marrow biopsy, compatible with FDG‐negative lymphoma (Table 1).

Treatment was documented in 62 patients. All received standard‐dose CHOP, with the addition of rituximab in CD20‐positive lymphomas (Table 1). Treatment according to the PETAL protocol comprised a minimum of six cycles of (R‐)CHOP. In the baseline PET‐negative group, six cycles were more frequently delivered to patients below than to patients above the age of 60 years (90% *versus* 67%; *P* = .05).

### 3.2 Outcome in patients with completely resected lymphoma

**Figure 1:**
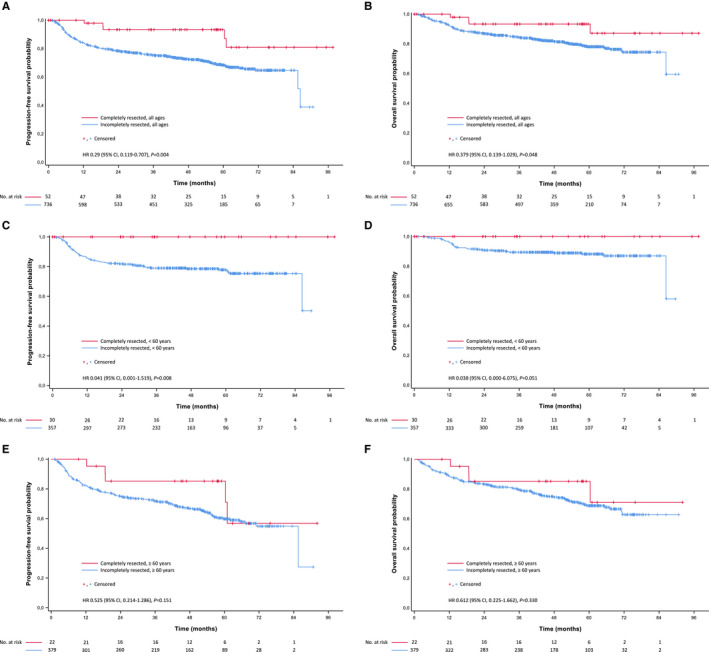
Kaplan‐Meier curves for (A, C, E) progression‐free survival and (B, D, F) overall survival in aggressive B‐cell lymphomas. (A, B) completely resected patients versus all patients from the PETAL comparator group. (C, D) completely resected versus incompletely resected patients < 60 years, and (E, F) ≥ 60 years, respectively

Patients with completely resected B‐cell lymphoma had significantly higher complete remission (100% versus 66.0%; *P* < .001) and overall response rates (100% versus 91.5%; *P* = .026) than B‐cell lymphoma patients with baseline PET‐positive disease participating in the main part of the PETAL trial (n = 736). With a median follow‐up of 4.4 years (interquartile range, 3.3‐5.4), progression‐free survival and overall survival were also superior in completely resected disease (Figure 1A,B). Because age is a strong risk factor, outcome was analyzed separately for patients below and above 60 years. The survival benefit for complete lymphoma resection was limited to the younger age group (Figure 1C‐F).

**Figure 2:**
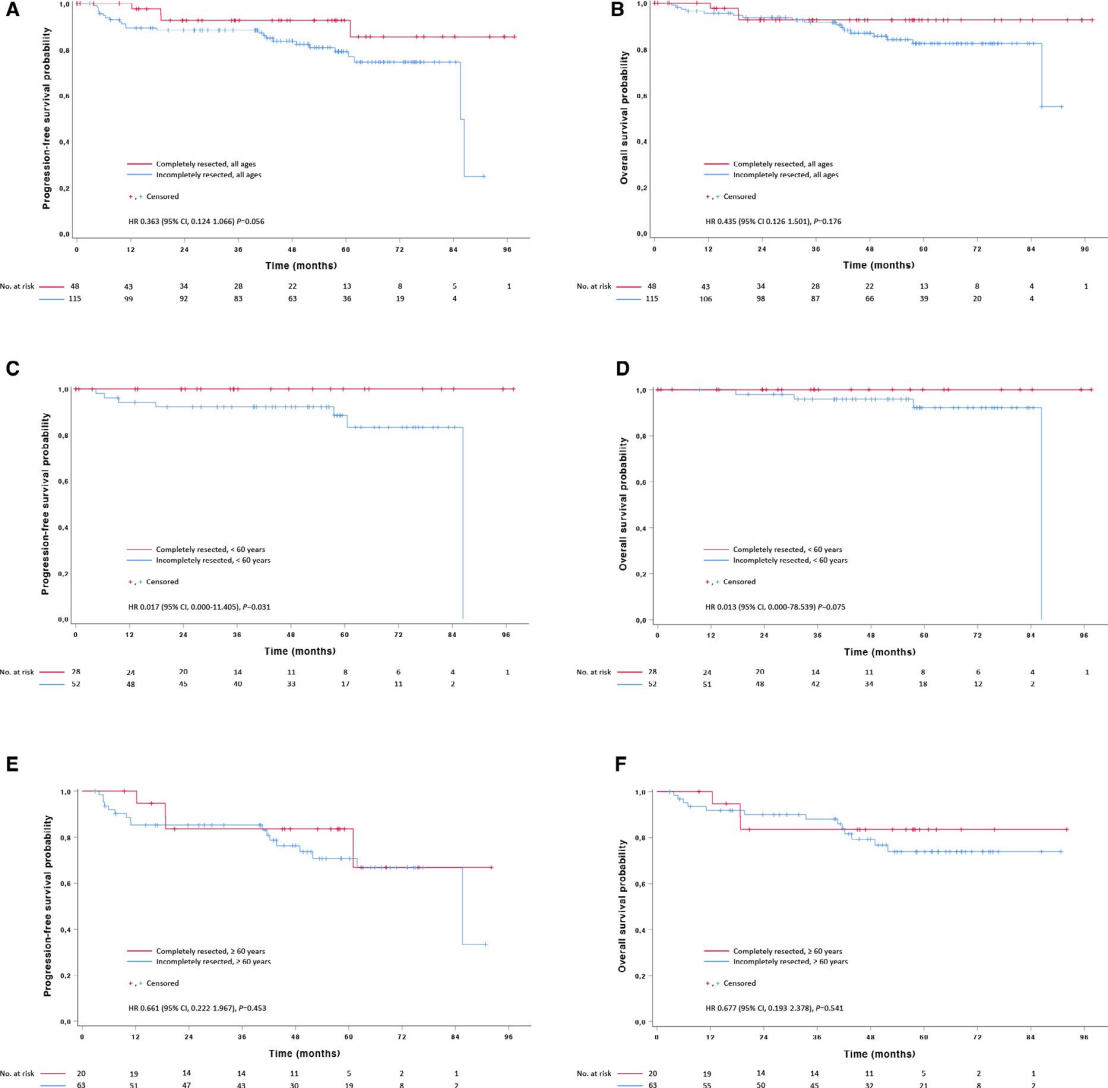
Kaplan‐Meier curves for (A, C, E) progression‐free survival and (B, D, F) overall survival in stage I diffuse large B‐cell lymphoma. (A, B) completely resected patients versus all patients from the PETAL comparator group. (C, D) completely resected versus incompletely resected patients < 60 years, and (E, F) ≥ 60 years, respectively

Without complete resection, baseline PET‐negative patients would have been classified as stage I disease. To define the impact of resection more accurately, we limited our comparison to stage I patients with diffuse large B‐cell lymphoma (DLBCL), which was by far the most frequent diagnosis (Table 1). Baseline features of stage I DLBCL patients with or without complete lymphoma resection were comparable except for lactate dehydrogenase levels which were less frequently elevated in completely than incompletely resected patients, consistent with lower tumor burden (Table 2). There was a trend for improved progression‐free survival in completely resected patients who reached statistical significance in the age group below 60 years, accompanied by a strong trend for improved overall survival. No such effects were seen in patients above 60 years (Table 2, Figure 2).

**Table 2:** Baseline characteristics and treatment outcome of patients with completely resected diffuse large B‐cell lymphoma in comparison to incompletely resected stage I patients from the PETAL cohort

	Completely resected lymphoma	Incompletely resected lymphoma	*P*
No. of stage I patients	48	115	
Baseline characteristics			
Age ≥ 60 years	20 (41.7%)	63 (54.8%)	.169
Male sex	33 (68.8%)	77 (67.0%)	.857
ECOG performance status ≥ 2	0 (0.0%)	3 (2.8%)	.557
Lactate dehydrogenase > ULN	5 (10.4%)	32 (27.8%)	.015
Superficial lymphoma location	36 (75.0%)	49 (42.6%)	<.001
Treatment outcome			
Complete remission rate	44/44 (100.0%)	89/108 (82.4%)	.02
Overall response rate	44/44 (100.0%)	106/108 (98.1%)	1.0
2‐year progression‐free survival (95% CI)			
All ages	92.7% (84.7‐100)	88.4% (82.5‐94.3)	.056
<60 years^a^	100% (n.a.)	92.2% (84.8‐99.6)	.031
≥60 years^a^	83.6% (66.5‐100)	85.3% (76.5‐94.1)	.453
2‐year overall survival (95% CI)			
All ages	92.7% (84.7‐100)	93.7% (89.2‐98.2)	.176
<60 years^a^	100% (n.a.)	95.9% (90.2‐100)	.075
≥60 years^a^	83.6% (66.5‐100)	90.1% (82.5‐97.7)	.541

Abbreviations: CI, confidence interval; ECOG, Eastern Cooperative Oncology Group; PETAL, Positron Emission Tomography‐guided Therapy of Aggressive non‐Hodgkin Lymphomas; ULN, upper limit of normal.

Response rates are given as number of patients responding/total number of patients with available data.

^a^ For patient numbers, refer to No. at risk in Figure 2.

**Figure 3:**
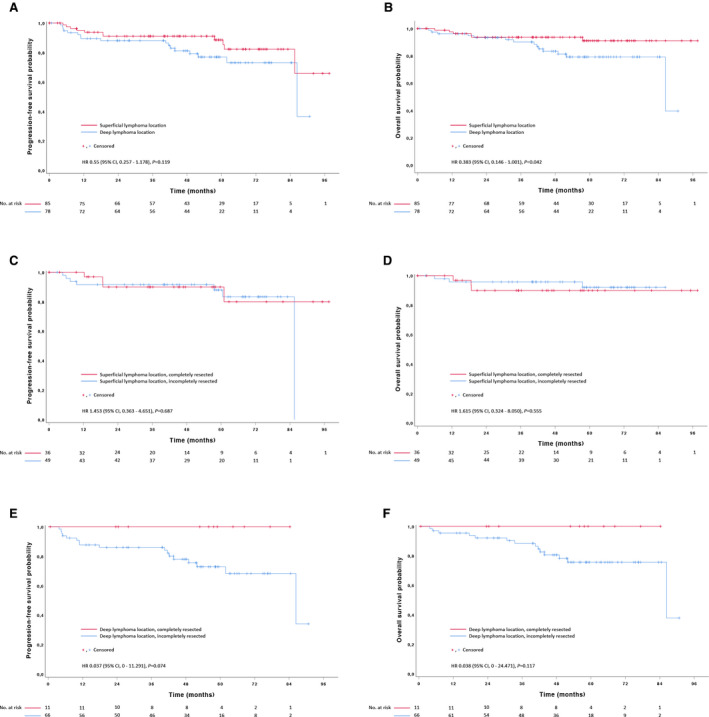
Kaplan‐Meier curves for (A, C, E) progression‐free survival and (B, D, F) overall survival in stage I diffuse large B‐cell lymphoma. (A, B) lymphomas in superficial versus deep location in completely and incompletely resected patients combined. (C, D) completely resected versus incompletely resected patients with superficial lymphoma location, and (E, F) deep lymphoma location, respectively

Superficial lymphoma locations were found in 75% of patients with completely resected disease, as compared to 42.6% in the control group (Table 2). Compared to deep locations, superficial locations were associated with significantly better overall survival and a trend for improved progression‐free survival (Figure 3A‐B). Compared to the control group of incompletely resected patients, complete resection of superficial lymphomas had no apparent impact on outcome, while deep locations appeared to benefit from resection (Figure 3C‐F). This finding did not reach statistical significance.

Because of small numbers, multivariable analysis was restricted to the entire group of stage I DLBCL patients. The only factor with statistically significant impact on progression‐free survival and overall survival was age. Complete lymphoma resection was associated with a weak trend for improved progression‐free survival (Table 3).

**Table 3:** Multivariable analysis for stage I diffuse large B‐cell lymphoma

	Progression‐free survival		Overall survival	
	HR (95% CI)	*P*	HR (95% CI)	*P*
Age ≥ 60 years	2.752 (1.161‐6.521)	.021	3.448 (1.145‐10.389)	.028
Female sex	0.537 (0.216‐1.336)	.181	0.281 (0.073‐1.074)	.063
ECOG > 1	2.557 (0.0.306‐21.351)	.386	5.405 (0.581‐50.323)	.138
LDH > ULN	1.268 (0.515‐3.122)	.606	1.004 (0.320‐3.153)	.995
Complete resection	0.461 (0.154‐1.380)	.166	0.547 (0.155‐1.931)	.348

Abbreviations: CI, confidence interval; ECOG, Eastern Cooperative Oncology Group performance status; HR, hazard ratio; LDH, lactate dehydrogenase; ULN, upper limit of normal.

FDG‐PET‐based tumor burden measurements were available in 103 of 115 patients with incompletely resected stage I disease. The median metabolic tumor volume of the remaining lymphoma was 30 cm^3^ (interquartile range, 10‐57).

**Figure 4:**
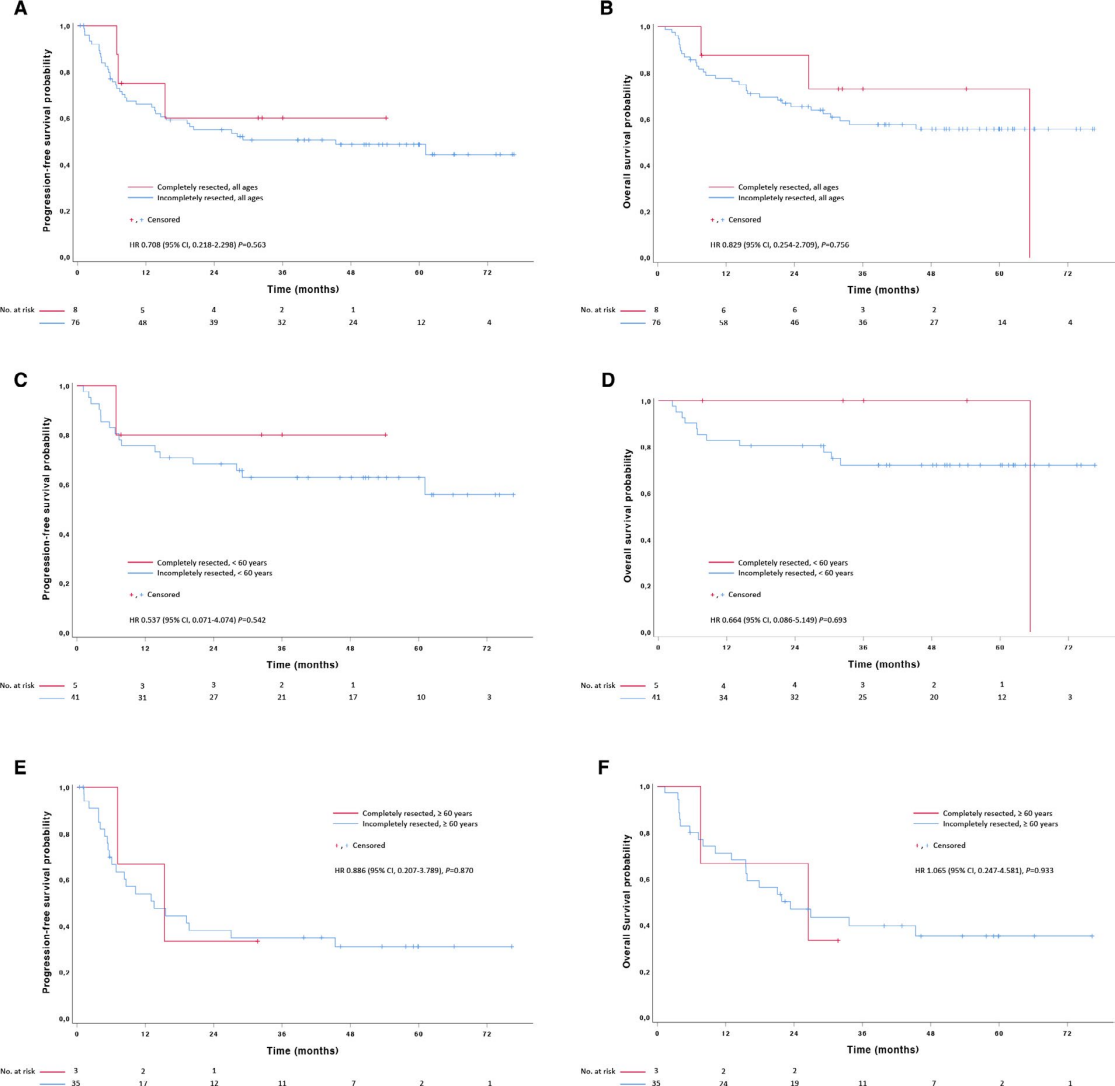
Kaplan‐Meier curves for (A, C, E) progression‐free survival and (B, D, F) overall survival in T‐cell lymphomas. (A, B) completely resected patients versus all patients from the PETAL comparator group. (C, D) completely resected versus incompletely resected patients <60 years, and (E, F) ≥60 years, respectively

In T‐cell lymphoma, no significant outcome differences were observed between patients with completely resected lymphoma and baseline PET‐positive patients treated in the main part of the PETAL study (N = 76). This was irrespective of age (Figure 4). Similar observations were made when the comparator group was confined to stage I patients (data not shown).

### 3.3 Outcome in baseline PET‐negative patients with pathological CT findings or a positive bone marrow biopsy

Four DLBCL and three T‐cell lymphoma patients with a negative baseline PET scan had findings compatible with FDG‐negative lymphoma (Table 1). In DLBCL, pathological lymph nodes were only slightly enlarged, the most frequent locations (1 patient each) being axillary (longest diameter, 17 mm), hilar (17 mm), and interaortocaval (24 mm). One DLBCL patient had histologically proven bone marrow infiltration not visualized on baseline PET. In T‐cell lymphoma, one patient (with angioimmunoblastic T‐cell lymphoma) had a mediastinal lymph node measuring 42 mm in length in addition to axillary and abdominal lymph nodes (16 mm), another (anaplastic lymphoma kinase‐negative anaplastic large‐cell lymphoma) had an inguinal lymph node measuring 21 mm, and the third (unclassifiable T‐cell lymphoma) had a positive bone marrow biopsy in addition to a completely resected cutaneous manifestation. None of the enlarged lymph nodes was biopsied.

Among the four patients with B‐cell lymphoma, complete remission rate was 100%, with predicted 2‐year progression‐free and overall survival rates of 100%. One of the three T‐cell lymphoma patients achieved an ongoing complete remission, while the other two progressed. At last follow‐up, all were alive.

## 4 DISCUSSION

Tumor burden is one of the strongest prognostic factors in aggressive lymphoma.^11^, ^12^ Because complete resection minimizes tumor mass, it could have a positive impact on outcome. In our study, this appeared to be the case in B‐cell, but not in T‐cell lymphoma. In B‐cell lymphoma, the benefit of complete resection was confined to patients below the age of 60 years. Possible explanations include increasingly unfavorable lymphoma features with increasing age,^13^ age‐related postoperative complications delaying the start of chemotherapy, and inconsistent treatment delivery in older patients.

The literature on the value of surgical debulking in aggressive lymphoma is scant and limited to retrospective studies. Romaguera et al^14^ reported a matched pair analysis of 28 stage I/II DLBCL patients treated with CHOP, bleomycin, and radiotherapy. Overall survival at 7 years was significantly better in patients undergoing debulking surgery (93% *versus* 35%). This finding was confirmed by Wilder et al^15^ who performed a case‐control study of 50 stage I/II aggressive lymphoma patients treated with CHOP and radiotherapy. At 5 years, local control rate (96% *versus* 80%), progression‐free survival (88% *versus* 62%), and overall survival (83% *versus* 71%) were better after debulking than after chemoradiotherapy alone. Kim et al^16^ focused on intestinal (mainly ileocecal) DLBCL treated with six cycles of CHOP or R‐CHOP with or without prior surgical debulking. In 250 stage I/II patients, surgery was associated with a significant improvement in the rates of complete remission (85% *versus* 64%), 3‐year progression‐free survival (82% *versus* 52%), and 3‐year overall survival (91% *versus* 62%), which was confirmed by multivariable analysis. There was no effect on outcome in more advanced stages. Finally, Bairey et al^17^ reported on the value of splenectomy in 87 patients with splenic DLBCL who were treated with (immuno‐)chemotherapy with or without radiotherapy. In stage I/II disease, splenectomy was associated with significantly improved progression‐free survival (at 5 years, 85% vs 55%) and overall survival (91% vs 68%), but, again, such an effect was not seen in more advanced stages.

These data together with our current analysis suggests that DLBCL patients may benefit from lymphoma resection before receiving curative immunochemotherapy, especially at young age and in early disease stages. Whether lymphomas in deep locations derive greater benefit from complete resection than lymphomas near the body surface requires confirmation in a larger study. Seventy‐five percent of patients in the stage I comparator population had a metabolic tumor volume <60 cm^3^. In many locations, tumors of such a size should be resectable without detriment. A welcome by‐product of complete lymphoma resection is obtaining a large tissue sample for the pathologist. In a retrospective study including 1,510 unselected patients, core needle biopsy failed to yield an unambiguous lymphoma diagnosis more frequently than excisional biopsy did (8.3% versus 2.8%).^1^ The success rate of core needle biopsy for lymphoma subtyping and grading has consistently been reported to be of the order of only 85%.^18^, ^19^, ^20^

Lymphoma resection did not improve outcome in peripheral T‐cell lymphoma. Because of small numbers the results have to be interpreted with caution. Similar to DLBCL, tumor mass is also a prognostic factor in T‐cell lymphoma.^21^ However, while in DLBCL tumor mass is a stronger predictor of outcome than treatment response determined by interim PET scanning,^4^, ^11^ the reverse appears to be true for T‐cell lymphoma.^12^ The proportion of chemotherapy‐refractory patients is much higher in T‐cell than B‐cell lymphoma,^5^ which may explain why treatment response is the overriding prognostic factor. Another factor that may have contributed to the results in T‐cell lymphoma is enteropathy‐associated T‐cell lymphoma, which was seen in three of 11 patients with completely resected disease *versus* none of 76 patients treated in the control group.^5^ Enteropathy‐associated T‐cell lymphoma has particularly poor outcome.^22^

In the majority of patients, a negative baseline PET scan appeared to be the result of complete lymphoma resection. Whether pathological CT findings in DLBCL patients with a negative baseline PET scan were indicative of FDG‐negative lymphoma appeared questionable. The size of the pathological lymph nodes was only slightly above normal, and they were found in locations where inflammatory reactions are frequent, such as axilla or lung hilum. Outcome of the affected patients was excellent. The true proportion of FDG‐negative DLBCL may therefore be lower than suggested by our study. This assumption is in line with previous investigations.^23^, ^24^, ^25^ In contrast to aggressive B‐cell lymphoma, FDG avidity is not always increased in T‐cell lymphoma.^23^, ^24^, ^25^ Glucose uptake varies from entity to entity, with particularly low values in peripheral T‐cell lymphoma, not otherwise specified, and angioimmunoblastic T‐cell lymphoma.^12^ In our study, at least one of the baseline PET‐negative T‐cell lymphoma patients (suffering from angioimmunoblastic lymphoma) had considerable lymph node enlargement suggestive of true FDG‐negative disease. Our study also highlights the limited sensitivity of baseline PET to detect bone marrow infiltration.^8^ One patient each with B‐cell or T‐cell lymphoma had histologically proven bone marrow disease escaping visualization by PET.

Strengths of our study include its prospective nature and the availability of a contemporaneous control group. There are also limitations. First, information about the reason for and the extent of complete resection was not available. Unlike solid tumors, macroscopic or microscopic disease along the tumor margins is generally not documented in lymphoma. Second, the assumption of complete resection relied on a single tumor biopsy, a single PET scan performed after surgery, and a bone marrow biopsy. Possible additional lymph nodes of borderline size were not biopsied, because the presence or absence of lymphoma would not have changed treatment. For the same reason, only two thirds of patients underwent bone marrow biopsy. Third, most patients with completely resected disease had superficial lymphomas. In our study, such lymphomas had better outcome than lymphomas in deep locations. In the control group, superficial and deep lymphomas were equally frequent. This may have led to overestimation of the beneficial effect of resection. Fourth, the impact of other prognostic factors, such as genetic abnormalities or gene expression, was not analyzed because these data were not available. Finally, our multivariable analysis failed to show a statistically significant influence of resection on outcome, which may have been related to the small sample size.

To our knowledge, the current analysis is the first prospective study in completely resected aggressive lymphoma. It confirms a possible benefit of resection in early stage DLBCL previously observed in retrospective studies. Complete resection indicates minimal tumor mass, which offers the opportunity to reduce curative therapy to four cycles of R‐CHOP, as recently shown in the FLYER trial.^11^ Our results lend support to the hypothesis that the prognosis of low‐tumor burden DLBCL may be further improved by complete lymphoma resection. When the presenting findings are compatible with the diagnosis of lymphoma and the affected area can be removed without jeopardy, complete resection could be worth a consideration.

## CONFLICT OF INTEREST

UD: Institutional research funding and honoraria from Roche and Amgen; AH: honoraria from Takeda and Celgene; travel reimbursement from Roche, Celgene, and Takeda, outside the submitted work. All the remaining authors have declared no conflict of interest.

## AUTHOR CONTRIBUTIONS

Christine Schmitz and Jan Rekowski involved in analysis and interpretation of the data, and drafting of the article. Stefan P. Müller conceptualized and designed the study, and involved in acquisition, analysis, and interpretation of data. Navid Farsijani, Bernd Hertenstein, Christiane Franzius, Ulla von Verschuer, Paul La Rosée, Martin Freesmeyer, Stefan Wilop, Thomas Krohn, Aruna Raghavachar, Arnold Ganser, Frank M. Bengel, Gabriele Prange‐Krex, Frank Kroschinsky, Jörg Kotzerke, and Aristoteles Giagounidis involved in acquisition, analysis, and interpretation of data. Ulrich Dührsen and Andreas Hüttmann conceptualized and designed the study, and involved in acquisition of funding, analysis and interpretation of the data, and drafting of the article.

## Data Availability

The data that support the findings of this study are available from the corresponding author upon reasonable request.
